# Regulation of Lipid Specific and Vitamin Specific Non-MHC Restricted T Cells by Antigen Presenting Cells and Their Therapeutic Potentials

**DOI:** 10.3389/fimmu.2015.00388

**Published:** 2015-07-28

**Authors:** Mariolina Salio, Vincenzo Cerundolo

**Affiliations:** ^1^MRC Human Immunology Unit, Weatherall Institute of Molecular Medicine, Radcliffe Department of Medicine, University of Oxford, Oxford, UK

**Keywords:** CD1, MR1, innate and adaptive immunity, lipids, vitamins

## Abstract

Since initial reports, more than 25 years ago, that T cells recognize lipids in the context on non-polymorphic CD1 molecules, our understanding of antigen presentation to non-peptide-specific T cell populations has deepened. It is now clear that αβ T cells bearing semi-invariant T cell receptor, as well as subsets of γδ T cells, recognize a variety of self and non-self lipids and contribute to shaping immune responses via cross talk with dendritic cells and B cells. Furthermore, it has been demonstrated that small molecules derived from the microbial riboflavin biosynthetic pathway (vitamin B2) bind monomorphic MR1 molecules and activate mucosal-associated invariant T cells, another population of semi-invariant T cells. Novel insights in the biological relevance of non-peptide-specific T cells have emerged with the development of tetrameric CD1 and MR1 molecules, which has allowed accurate enumeration and functional analysis of CD1- and MR1-restricted T cells in humans and discovery of novel populations of semi-invariant T cells. The phenotype and function of non-peptide-specific T cells will be discussed in the context of the known distribution of CD1 and MR1 molecules by different subsets of antigen-presenting cells at steady state and following infection. Concurrent modulation of CD1 transcription and lipid biosynthetic pathways upon TLR stimulation, coupled with efficient lipid antigen processing, result in the increased cell surface expression of antigenic CD1–lipid complexes. Similarly, MR1 expression is almost undetectable in resting APC and it is upregulated following bacterial infection, likely due to stabilization of MR1 molecules by microbial antigens. The tight regulation of CD1 and MR1 expression at steady state and during infection may represent an important mechanism to limit autoreactivity, while promoting T cell responses to foreign antigens.

## Introduction

Earlier studies in the 1990s demonstrated that the antigen recognition potential of T lymphocytes is not limited to peptides presented by MHC class I and class II molecules (1, 2). Indeed, the newly identified MHC-related genes belonging to the CD1 family (3) were soon shown to present self and mycobacterial lipids to αβ and γδ T cell clones lacking CD4 and CD8 co-receptors (1, 2, 4). Furthermore, human and murine T cells bearing semi-invariant T cell receptors (TCRs) (5, 6) were shown to be CD1d restricted (7).

A second MHC-related gene was identified in 1995, MR1 (8), which in 2003 was shown to select a population of cells known as mucosal-associated invariant T cells (MAIT) (9), also bearing semi-invariant TCRs (10). It was not until 2012, however, that microbial vitamin B2 metabolites were identified as the elusive antigens presented by MR1 molecules (11).

In the past 25 years, a number of investigators have elucidated the contribution of CD1- and MR1-restricted T cells to antimicrobial immunity, and for CD1-restricted T cells also to cancer immune-surveillance and autoimmunity. While comprehensive reviews on CD1 and MR1 antigen-presenting systems have been recently published (12–15), we will focus on recent findings that have advanced our understanding of the role of CD1- and MR1-restricted T cells, also known as non-conventional T cells or innate-like cells, as they straddle between innate and adaptive immunity.

## CD1 Molecules

**Figure 1:**
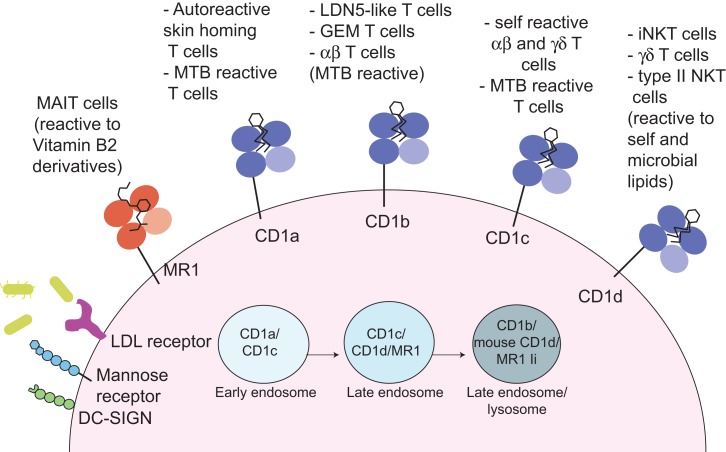
**Antigen presentation to non-peptide-specific T cells**. MR1 and CD1 molecules present vitamin B2 derivatives or self and microbial lipids to a variety of αβ or γδ-bearing T cells. Through a variety of receptors (such as DC-SIGN, mannose receptor, and LDL-receptors) or via phagocytosis (not depicted) antigen-presenting cells uptake incoming pathogens. Microbial antigens are distributed through the endocytic compartment where they intersect recycling MR1 and CD1 molecules. In these compartments, antigen loading occurs, often through the help of accessory molecules such lipid transfer proteins (not depicted). The invariant chain (Ii) facilitates MR1 distribution in the late endosomal/lysosomal compartments (27).

The human CD1 locus on chromosome 1 encodes five molecules, divided into group 1 (CD1a, b, and c) and group 2 (CD1d), based on sequence homology (3, 16). The fifth molecule, CD1e, is not expressed at the cell surface, yet plays an important role in assisting lipid antigen processing and loading on group 1 CD1 molecules (17). CD1 molecules are heterodimers of a heavy chain non-covalently associated with β-2 microglobulin, and have an overall fold similar to MHC class I molecules, however, unlike MHC class I and class II molecules, they are not polymorphic (3, 16). In comparison to MHC class I molecules, CD1 molecules have evolved a deep and narrow binding cavity that anchors the hydrophobic alkyl chains of lipid molecules: the binding cavity contains two pockets, A′ and F′, of which the A′ is deeper and closed by a narrow entrance at the top. Yet, each CD1 molecule differs in the antigen-presenting groove architecture, in the intracellular trafficking pattern, and in the overall tissue expression (18, 19). These differences underscore the non-redundant physiological role of the CD1 isoforms, which sample a variety of lipids in early, late endosomes or deep in the lysosomes, where exogenous lipids distribute according to their biophysical properties (20) (Figure 1). In mice, group 1 CD1 genes are absent and it is thought that they were lost during evolution, as they are present in other rodents (21). This has greatly hindered our understanding of the role and frequency of group 1 CD1-restricted T cells, until the recent development of CD1a, b, and c tetramers, which has opened the way toward enumeration and functional characterization of human lipid-specific T cells (22–24). Humanized SCID mice and group 1 CD1 transgenic mice are also proving to be useful models to study the role of CD1-restricted T cells in disease settings (25, 26).

### CD1a

CD1a molecules are expressed on double-positive thymocytes, while in the periphery their expression is restricted to tissue-resident dendritic cells (DCs) and Langerhans cells (LC) in the skin (28). Unlike other CD1 isoforms, CD1a molecules have a short cytoplasmic tail, with no tyrosine-based motif to drive their recycling through late endocytic compartments. Hence, their trafficking is limited to the early endosomal compartment in a Rab22- and Arf6-dependent manner (29). Of all the CD1 molecules, CD1a has the smallest groove, which is suitable to present antigens encountered in the early endosomal compartment or at the cell surface (30–32).

CD1a-restricted cells can be autoreactive or pathogen reactive. The only microbial antigen known to bind CD1a is the mycobacterial lipopeptide didehydroxymycobactin (DDM) and DDM-restricted T cells could play a pivotal role in early detection of *Mycobacterium tuberculosis* infection (33). Like for many other lipid-specific T cells, recognition is exquisitely sensitive to the structure of the peptide and to the length and saturation of the fatty acid, which influences the positioning of the peptide residues available for recognition by the TCR (31). Despite a low affinity interaction (100 μM) between a DDM-specific TCR and CD1a–DDM soluble molecules (23), DDM–CD1a dextramers have been successfully used to stain DDM-specific T cells *ex vivo* in patients with active tuberculosis or positive tuberculin test, and could be a useful tool to determine the phenotype and function of these cells at a population level (23).

The first ever reported CD1-restricted clone was self-reactive (1). One of the first identified self-antigens presented by CD1a is sulfatide, a glycolipid abundant in myelin sheets. Of note, sulfatide can also be presented by CD1b, CD1c, and CD1d (34), which suggested a possible contribution of CD1-restricted T cells to the autoimmune response in multiple sclerosis (MS). To further characterize the pool of CD1a-autoreactive T cells, Moody, and co-workers have recently designed an experimental system based on CD1-expressing human myelogenous leukemia cells (K562 cells), with low or absent expression of MHC molecules in order to limit allo-reactivity. These studies have demonstrated that polyclonal CD1a reactive T cells are present at high frequency in the peripheral blood of healthy individuals [0.02–0.4% of memory T cells (35, 36)]. Similar results were independently obtained with C1R cells as antigen-presenting cells, although in this case higher frequencies of CD1a (and CD1c) reactive cells were observed [up to 10% of circulating T cells (36)]. Interestingly, CD1a-restricted T cells found in the blood express the skin-homing receptors CLA, CCR6, CCR4, and CCR10 and produce the cytokine interleukin 22 (IL-22) in response to CD1a^+^ DCs. The identification of CD1a-restricted cells in skin biopsies suggests that they may be playing an important immunoregulatory role in skin homeostasis through IL-22 secretion (35). It will be very interesting to investigate whether they may also play a role in skin immunopathology in psoriasis or in other skin diseases where over production of IL-22 has been implicated (37).

To understand the nature of the antigens activating CD1a-restricted T cells, self-ligands were eluted from secreted CD1a molecules and skin samples and tested *in vitro* (38). Unexpectedly, stimulatory antigens were more efficiently extracted in chloroform than in the commonly used chloroform methanol mixture, suggesting high hydrophobicity. Indeed, CD1a molecules were found to stimulate T cell clones when loaded with oily antigens lacking carbohydrate or charged head groups [such as triacylglyceride (TAG), squalene, and wax esters], while lipids with hydrophilic head groups inhibited CD1a-restricted T cell autoreactivity (38). These results, which suggested a unique mode of “headless” antigen recognition by autoreactive CD1a-restricted T cells, were recently confirmed and extended with structural and mutagenesis studies (39). Although two of the studied autoreactive TCRs have binding affinities for CD1a–self complexes at the low end of the spectrum (30 and 93 μM (38, 39), CD1a tetramers loaded with a spectrum of permissive ligands [such as phosphatidylcholine and lysophosphatidylcholine (LPC)] have been shown to stain Jurkat cells transduced with one of these TCR (39). Furthermore, the ternary structure of two TCR–CD1a–self-lipid complexes showed that the TCR docks over the A′ roof of CD1a molecules without direct contact with the antigenic ligand. A comparison of these structures with those of CD1a–sulfatide (30) or CD1a–lipopeptide (31) provided a molecular explanation for the inhibitory effect of polar ligands, which are thought to disrupt the TCR–CD1a contact zone (39), revealing a mode of antigen recognition different from TCRs of peptide-specific T cells and other CD1-restricted T cells, centered on critical interactions with antigens bound to MHC or CD1 molecules (40).

TAG, fatty acids, and squalene accumulate in sebaceous glands and in the corneous stratus of the epithelium, separated from epidermal LC. So it is likely that at steady state, LC will not efficiently load these stimulatory antigens on CD1a molecules. However, upon trauma, infection or any form of barrier breach, these antigens could gain access to LC and increase the response of CD1a-autoreactive T cells. Consistent with this hypothesis, recently, CD1a-restricted responses have been documented in cohorts of patients allergic to bee and wasp venom (41). Despite the high lipidic content of wasp and bee venoms, in these patients the culprit antigens are not exogenous, but are generated *in vivo* by venom phospholipase A2 injected intradermally by wasps and bees via their sting (41). *In vitro*, it was shown that venom phospholipase A2 activates CD1a-restricted T cells cleaving non-antigenic phospholipids into lysophospholipids and antigenic headless fatty acids. Using an *in vivo* model of suction cap blisters, by mass spectrometry the authors also demonstrated the presence of lysophospholipids in the blister fluids of volunteers injected with venom. Although free fatty acids were not detected in the blister fluids, is it likely that this negative result was due to lack of sensitivity of the mass spectrometry (41).

Thus, the physical separation between antigen and antigen-presenting cells and/or the balance between stimulatory and inhibitory CD1a-ligands seem to be two of the mechanisms that the immune system deploys to keep an abundant population of autoreactive T cells under control at steady state. As endogenous or exogenous phospholipases can be activated during exposure to several allergens, it will be of interest to investigate whether in cohorts of patients with atopic dermatitis similar mechanisms may be active and account for expansion and/or activation of autoreactive T cells.

The identification of phospholipase A2 as a novel mechanism to generate autoantigens may offer new diagnostic and therapeutic opportunities. Likewise, as immunostimulatory oils and hydrocarbons are components of widely used adjuvants such as MF59, it will be important to address the role of CD1a-autoreactive T cells in shaping the adaptive T cell response during the aforementioned vaccination protocols.

### CD1b

CD1b molecules are expressed on thymocytes and on peripheral DCs. Through tyrosine-based cytoplasmic motifs CD1b molecules bind to both AP-2 and AP-3 adaptors (42, 43) and efficiently traffic to acidic LAMP1^+^ lysosomes, where processing of complex lipid antigens may occur and loading is aided by the acidic pH and by lipid transfer proteins (17, 44, 45). Additionally, CD1b molecules have evolved the largest antigen-presenting groove, with three pockets (A′, C′, and F′) and a large tunnel, which can accommodate lipids with very long alkyl chains, such as mycobacterial antigens with up to 80 carbons (46, 47). As most cellular lipids do not exceed 40 carbons length, the architecture of the groove of nascent CD1b molecules is maintained by spacer (or scaffold) lipids, such as diacylglycerols and deoxyceramides (48, 49). The existence of spacer lipids was initially suggested from the crystal structures of CD1b bound to the ganglioside GM2 or phosphatidylinositol, where it was observed that detergent moieties occupied the channels not filled by the lipid ligands (46). Spacer lipids seat at the bottom of the antigen-presenting groove, providing support for antigens loaded in the CD1b molecule, and are displaced when loading of longer microbial lipids occurs (49, 50). Furthermore, by stabilizing the antigen-presenting molecules, they enhance presentation of microbial lipids with shorter acyl chains (49). Spacer lipids have also been found when CD1c and CD1d molecules were crystallized with short lipids (51, 52), so their use seems to be a common strategy to maintain the correct fold and antigen orientation for CD1 molecules.

While CD1b-autoreactive T cells have been described (53, 54), they are detected at lower frequency than for other CD1 members (35). Indeed, CD1b molecules are specialized in presenting bacterial antigens, perhaps because of the large volume of the groove; so far the majority of the described ligands are of mycobacterial origin. Mycolic acid from *M. Tuberculosis* cell wall was the first described lipid antigen presented by CD1 molecules (4), and it can form the scaffold for other mycolyl antigens, such as glucose monomycolate (GMM) and glycerol monomycolate (55). Other families of CD1b lipid antigens are derivatives of phosphatidyl-myo-inositol (such as phosphatidylinositol mannoside (PIM) and lipoarabinomannan (LAM)) and sulfoglycolipids. The structure–activity relationship of these classes of lipids has been recently reviewed in Ref. (56, 57) and we refer the reader to these excellent reviews for more detail.

The development of CD1b tetramers has recently allowed to track mycobacteria-specific CD1b-restricted T cells in the blood of individuals with active tuberculosis or previous *M. tuberculosis* (MTB) exposure (22). In addition to following the dynamics of lipid-specific immune responses, for those lipid antigens produced only by *M. tuberculosis* and not other mycobacterial species (such as sulfoglycolipids), tracking of antigen-specific T cells may represent an important future tool for differential diagnosis.

Until now, it was assumed that group 1 CD1-restricted T cells were expressing highly diverse TCR, like peptide-specific MHC-restricted T cells. However, the use of tetramers to study the CD1b-GMM-specific T cell response in multiple individuals has allowed the discovery of two novel T cell types in the human repertoire, germ-line encoded mycolyl reactive (GEM) T cells (58) and LDN5-like T cells (59), which stain brightly and dimly, respectively, as indication of higher (around 1 μM) and lower affinity (20–40 μM) for CD1b–GMM complexes. GEM T cells express a semi-invariant TCR using TRAV1.2 variable segments rearranged to TRAJ9 joining segments (thus differing from the TRAV1.2-TRAJ33 TCR used by MAIT cells, see later), with nearly identical CDR3 sequences and a biased TRBV6.2 usage. LDN5-like T cells have a biased TRAV17 usage, with uniform CDR3 length, and a biased TRBV4.1 usage, with variable CDR3 length. Structural data point to a role of the TCR-β chain in influencing the fine specificity of the GMM-specific TCRs (58). The evidence to date suggests that GEM and LDN5-like T cells expansion is antigen driven upon infection, and that they do not represent another population of innate-like cells, unlike the semi-invariant MAIT and iNKT cells; however, a detailed transcriptional and functional analysis of these cells is yet to be done. Furthermore, it still remains to be determined whether GEM and LDN5-like T cells show immunological memory, how long they persist, and whether they can be harnessed for vaccination purposes. Finally, it remains to be determined what drives the selection of these cells in donors with no documented mycobacterial infection.

### CD1c

CD1c molecules are expressed on thymocytes and at high density on peripheral DCs, LC (together with CD1a), and B cells. Through associations with AP2 adaptor molecules, they are widely distributed through the endocytic system (but not the LAMP1^+^ lysosomes), which allows sampling of a broad spectrum of lipids in a variety of antigen-presenting cells (60).

CD1c molecules present self and microbial lipids to T cells bearing αβ and γδ TCR. In 1989, Porcelli and colleagues demonstrated specific recognition of CD1c by a CD4^−^CD8^−^ γδ CTL line (1) and CD1c self-reactivity was later confirmed with other cytotoxic γδ lines bearing the Vδ1 segment (61, 62). The self-antigens recognized by these CTL lines, though, still need to be identified.

CD1c-autoreactive αβ T cells are present at high frequency in the peripheral blood of healthy donors (36). Recently, a novel self-lipid antigen (methyl-lysophosphatidic acid, mLPA) that accumulates in leukemic cells has been identified as one of the targets of CD1c-reactive T cells (63). mLPA-specific T cell clones were shown to efficiently kill *in vitro* and *in vivo* primary leukemia cells in a CD1c-restricted manner, but not normal B cells and primary DC, that despite being CD1c positive do not express the antigen at significant level (63). Selective accumulation of mLPA in human leukemia suggests that it can be considered a novel class of tumor-associated antigens and may represent a promising immunotherapeutic target.

CD1c molecules present several mycobacterial and synthetic lipids with methylated alkyl chains: mannosyl phosphodolichols (MPDs), mannosyl-β1-phosphomycoketide (MPM), and phosphomycoketide (PM) (24, 64–66). The mycobacterial enzyme polyketide synthase 12 (psk12) is crucial for the synthesis of the methyl-branched lipids, which are a molecular signature of mycobacterial infection and essential for antigenicity (24, 65). Polyclonal CD1c-restricted T cells expand *in vivo* during mycobacterial infection and can be tracked with lipid-loaded CD1c tetramers (24, 64).

The range of self and foreign antigens presented by CD1c may be larger than currently appreciated, as it has been shown that also lipopeptides can be antigenic, in analogy to CD1a. This was demonstrated as a proof of principle with a synthetic *N*-acyl glycine dodecamer lipopeptide (lipo-12) (67). These results raise the possibility that other eukaryotic or viral *N*-terminally acylated peptides, such as post-translationally modified products of ribosomal translation, might be antigenic. Interestingly, as lipopeptide presentation is sensitive to proteolysis in late endosomes and lysosomes (67), CD1c and CD1a molecules are the two CD1 isoforms uniquely suited to lipopeptide presentation because they predominantly survey the secretory pathway and the early endosomes.

Structural studies have highlighted a partially open structure of CD1c F′ pocket (52), which may accommodate a variety of ligands, from diacylated lipids such as sulfatide (34), to lipopeptides and possibly aid antigen loading in the early endosomal compartment or at the cell surface, in the absence of specific lipid transfer proteins. Furthermore, the CD1c–MPM crystal structure has highlighted the essential role for the methyl branches of MPM in stabilizing the single alkyl chain in the A′ pocket (52). Finally, the exquisite specificity of MPM and PM reactive clones can also be explained at the structural level (68): in the absence of the mannosyl moiety, the phosphate head group of PM is shifted toward the F′ pocket. A range of TCR binding affinities (7–30 μM) have been reported for CD1c–mycoketide complexes, and while the ternary complex TCR–CD1c–antigen is not yet available, biophysical data with six CD1c-reactive TCRs showed that different TCRs used different docking strategies on the same CD1c–lipid complex, unlike what has been described for the iNKT TCR and is predicted for the GEM TCR (68).

### CD1d

CD1d molecules are the most widely distributed, as they are expressed not only on hematopoietic cells (thymocytes, monocytes, DCs, and B cells), but also on epithelial cells (28). CD1d-restricted T cells are collectively known as natural killer T cells, because of co-expression of T cells and NK cell markers (most notably CD161 in humans and NK1.1 in some mouse strains). Two types of NKT cells exist: type I, (also known as invariant, iNKT), expressing a semi-invariant TCR (Vα24-Jα18 paired to Vβ11 in humans, Vα14-Jα18 paired to Vβ2, Vβ7, or Vβ8.2 chains in mice); type II, expressing a polyclonal TCR repertoire (12). The CD1d antigen presentation system is conserved across species, and both human and murine iNKT cells can be tracked with CD1d tetramers loaded with the synthetic glycolipid agonist α-galactosylceramide (α-GalCer). Furthermore, the availability of murine models lacking type I or type I and II NKT cells has greatly contributed to our knowledge of the biology of these cells. Conversely, we still lack reagents to specifically detect the majority of type II NKT cells, thus with few exceptions, their role *in vivo* has been less characterized.

Through recognition of a variety of self and microbial antigens, NKT cells have an important immune-regulatory role, spanning from autoimmunity, to protection against infection and tumor immune-surveillance. We refer the reader to recent reviews for a comprehensive discussion of NKT cell biology and CD1d antigen presentation (12, 14, 69–71), while here we highlight the role of microbiota in modulating iNKT cell reactivity and we summarize results revealing a previously unknown heterogeneity of the human NKT cell family (Figure 1).

#### Microbiota and NKT Cells

Regulation of metabolism and immunity by commensal bacteria is now well established (72). Interestingly, α-GalCer, the most potent iNKT cell agonist to date, was originally isolated from commensal bacteria of the marine sponge *Agela mauritianus* (73). The α-anomeric linkage of the sugar moiety is the quintessence of a microbial signature, and a variety of iNKT cell agonists from different microbial species have been characterized, although during microbial infection iNKT cell reactivity is often driven by cytokine-mediated signals (74). Recently, inhibitory and activatory α-GalCer species have been biochemically isolated from *Bacteroides fragilis*, a prominent species of the gut microbiota (75, 76). It has also been demonstrated that the intestinal microbiota plays an important role in the tight regulation of iNKT cell numbers and function, possibly through the balance between stimulatory and inhibitory lipids: germ-free mice have increased relative and absolute numbers of iNKT cells in the intestine, due to increased CXCL16 expression in the mucosal epithelium and CXCL16-dependent iNKT cell homing (77). The conditioning effect of the microbiota starts very early in life and has long-lasting consequences, as demonstrated by higher susceptibility of germ-free mice to intestinal immunopathology and lung inflammation (77). In turn, iNKT cells influence bacterial colonization of the intestine and lungs of mice (78) and signaling through epithelial CD1d is essential in maintaining mucosal homeostasis via IL-10 secretion (79). These findings have been recently extended in humans, where phenotypically and functionally mature iNKT cells have been detected in the sterile environment of the fetal intestine, and it is thought that they may represent an important first line of defense at birth (80). Furthermore, lysosulfatide-reactive CD1d-restricted type II NKT cells have been identified in the mucosa of ulcerative patients, and their cytotoxic activity against the intestinal epithelium suggests a pathogenic role (81). It remains to be determined whether during intestinal inflammation, T cells restricted by group 1 CD1 may also recognize self or microbial lipids.

#### Human NKT Cell Heterogeneity

##### Adipose tissue-resident iNKT cells

Like MHC-restricted CD4 cells, iNKT cells can also differentiate in Th1, Th2, Th17, T_FH_, and T-regulatory subsets, which use the same transcription regulators as peptide-specific T cells (69). The balance between subsets could have profound regulatory effects during immune responses, through the secretion of cytokines and modulation of DC and B cell function (12). Recently, a tissue resident subset of iNKT cells with a unique transcriptional and cytokine profile has been shown to accumulate in adipose tissue and regulate the function of Tregs and macrophages, via IL-2 and IL-10, respectively (82). Adipose tissue iNKT cells do not express the master regulatory PLZF, but express the transcription factor E4BP4, which controls IL-10 production. Also, as compared to splenic or liver iNKT cells, a smaller fraction of adipose tissue iNKT cells expresses CD44 and NK1.1 markers, while expression of ICOS and PD-1 was increased. As adipocytes are CD1d positive, they could modulate iNKT cell activation through presentation of self and dietary lipids, and ultimately the cross talk between iNKT cells and adipose tissue macrophages could be very important in preventing tissue inflammation. This hypothesis is consistent with the described protective role of iNKT cells against obesity-induced chronic inflammation (83).

##### CD1d-restricted γδ T cells

Two groups employed CD1d tetramers loaded with two different ligands to isolate CD1d-reactive T cells from healthy human peripheral blood. In one study, the majority of CD1-sulfatide tetramer staining cells were found to be T cells bearing the Vδ1 TCR (84), while a second study identified Vδ1 T cells amongst those binding CD1d-α-GalCer tetramers, although the majority of cells binding to the latter tetramers, as expected, were iNKT cells (85). Interestingly, human Vδ1 do not recognize mouse CD1d-α-GalCer tetramers, unlike human iNKT cells, highlighting a clear difference in the reactivity of the two populations. Also the affinity of binding of Vδ1 cells to human CD1d-α-GalCer complexes is lower than that observed for iNKT cells (Kd 16 versus 0.5 μM).

Vδ1 bearing cells are typically tissue-homing γδ cells and are abundant in the intestinal mucosa (86, 87). Preliminary results suggest that some reactivity to C1R cells expressing CD1d molecules can be detected amongst Vδ1 polyclonal lines generated from intestinal biopsies (88). Given the presence of several lipids from the microflora and the abundant expression of CD1d on the gastrointestinal epithelium (28), future studies should investigate whether intestinal Vδ1 γδ T cells can also bind CD1d-α-GalCer tetramers and if so, the role of microbiota in maintaining and expanding Vδ1 γδ T cells after birth. As Vδ1 cells are present at higher frequency than iNKT cells, they could have a marked impact on intestinal homeostasis and immunopathology, and reactivity could be modulated by the expression of stress-induced MHC-related molecules like MICA and MICB (86). Likewise, reactivity to sulfatide may underscore a possible role of these cells in MS.

The mode of γδ TCR–CD1d-α-GalCer/sulfatide recognition is markedly different from that of the iNKT TCR (85, 88). The γδ TCR docks orthogonally rather than in a parallel manner like the iNKT TCR, thus resembling type II NKT TCRs and classical peptide-specific TCRs (40); CD1d binding is dominated by the TCRδ chain, while CDR3γ residues contribute to lipid antigen binding only in CD1d-α-GalCer, but not in CD1d–sulfatide ternary complexes (85, 88).

##### NKT cells and chronic inflammation

Several investigators have described reactivity of human type II NKT cells toward inflammation-associated lysolipids, generated by the action of PLA A2 (89). T cells binding CD1d–LPC multimers were found at higher frequency in the blood of myeloma patients compared to healthy controls, consistent with elevated serum levels of LPC in the plasma of these patients (90). In infected hepatocytes, Hepatitis B was shown to induce the activity of secretory phospholipases and the release of lysophosphatidylethanolamine (lyso PE), capable of eliciting CD1d-restricted type II NKT cells activation in humans and mice, suggesting that they may play a role in viral recognition (91).

Glucosylsphingosine (LGL1), the deacylated product of β-glucosylceramide (GL1), accumulates in several metabolic disorders such as Gaucher disease, as a consequence of altered sphingolipid metabolism. In all metabolic disorders, lipid accumulation is associated with progressive inflammation. One of the contributing factors could be the expansion of pathogenic LGL1-reactive CD1d-restricted type II NKT cells with a T_FH_ phenotype, stimulating inflammation and B cell activation (92).

The demonstration that lysolipid species are antigenic for subsets of CD1d-restricted NKT cells is of great interest and provides the link for NKT cell activation in sterile inflammatory conditions, possibly suggesting novel therapeutic modalities through selective inhibition of the biochemical pathways generating the relevant antigens.

#### On the Role of DC in Regulating CD1 Reactivity

The central role of DCs in orchestrating immune responses is now well established (93). Immature DC, residing in the periphery, patrol the body for incoming pathogens and recognition of pathogen molecular patterns (PAMPs) through pattern recognition receptors (PRRs) triggers DC activation, maturation, and migration to the draining lymph nodes. Coordinated changes in expression of MHC class I and II, co-stimulatory molecules and cytokines upon DC maturation, promote efficient priming of peptide-specific CD4, CD8 T, and B cells in the lymph node (94, 95). The heterogeneity in DC subsets and their different anatomical distribution results in unique functional specialization, and ensures tailoring the adaptive immune response to the type of incoming stimulus (95).

As highlighted in the previous paragraphs, CD1d expression is constitutive and shared by all DC subsets; however, expression of group 1 CD1 molecules is much more restricted. Due to the strong autoreactivity of CD1-restricted T cells, tight regulation of steady-state cell surface expression of CD1 is required to control their activation. For example, lipids found in human serum, particularly lysophosphatidic acid and cardiolipin, inhibit group 1 CD1 expression, through a transcriptional mechanism involving activation of the peroxisome proliferator-activated receptor (PPAR) nuclear hormone receptors (96).

Monocytes express only CD1d molecules, but during *in vitro* differentiation into DC with GM–CSF and IL-4, group 1 CD1 expression is induced (2). It is likely that, *in vivo*, cytokines in the local microenvironment might influence group 1 CD1 expression in the process of monocyte to DC differentiation following transendothelial migration (97). Indeed it has been shown that monocyte infection with mycobacteria represents an efficient way to induce DC differentiation and expression of group 1 CD1 molecules (98–100). Upregulation of CD1 molecules depends on NOD and TLR signals and is enhanced by concomitant inflammasome activation and release of bioactive IL-1β (101). Interestingly, while mycobacterial infection increases group 1 CD1 expression, it downregulates CD1d and interferes with MHC-restricted antigen presentation (99, 102).

Mycobacterial cell wall lipids thus have a dual effect, by serving as antigens (i.e., mycolic acids, GMM, LAM PMK, and DDM) and adjuvants that drive CD1 expression on the infected cells, to promote antigen presentation. However, group 1 CD1 molecules are not expressed on macrophages, which instead are the infected cells during *in vivo* MTB infection, thus whether CD1b-restricted T cells might play a sizeable cytotoxic and anti-mycobacterial function *in vivo* is debatable. The low frequencies of group 1 CD1-restricted T cells, even after infection, rather suggests a helper function, perhaps through modulation of DC function (103).

While during DC maturation a marked upregulation of MHC class I and II is observed, with increased half-life of surface MHC–peptide complexes leading to efficient peptide antigen presentation (104, 105), the surface expression of group 1 CD1 molecules is only moderately increased (CD1b, CD1c) or even decreased (CD1a) (106); furthermore, CD1 molecules continue to recycle between the plasma membrane and intracellular compartments (102). CD1-mediated lipid antigen presentation occurs very efficiently already in immature DC and this might ensure prompt licensing of DC by lipid-specific T cells via cytokines and CD40–CD40L interactions (103). The role of iNKT cells in DC licensing and memory CTL generation is discussed in depth in an accompanying review in this issue.

To ensure optimal antigen presentation through CD1 molecules, DC subsets also coordinate lipid antigen uptake and distribution through the endosomal compartment through specific receptor-mediated interactions (19). Serum lipoproteins ensure efficient delivery of self and foreign antigens for CD1-mediated presentation, through ApoE-LDL-R-mediated uptake (107).

The C-type lectin Langerin mediates *Mycobacterium leprae* antigen uptake and delivery to Birbeck granules in LC and is required for CD1a–lipid antigen presentation (108). The mannose receptor (CD206), a C-type lectin expressed on macrophages, dermal DC, and monocyte-derived DC, promotes mycobacterial LAM uptake and lysosomal delivery for CD1b presentation (109). Other C-type lectins that specifically capture pathogen-derived carbohydrate rich antigens are DEC 205 (expressed on LC, dermal DC, and monocyte-derived DCs), DC-SIGN (CD209, expressed mainly on dermal DC). Their role in enhancing peptide presentation is well described (110, 111) and it is likely that these and other related molecules involved in endocytosis of bacteria or bacterial debris might also influence CD1d-restricted lipid antigen presentation in late endosomal compartments. Selective expression of endocytic receptors in DC subsets can also be exploited therapeutically: recently it has been shown that targeted delivery of the mycobacterial antigen GMM to monocyte-derived DCs via Siglec-7 via sialic acid-coated nanoparticles induces robust CD1b-restricted T cell activation, although this was not tested on primary CD1b^+^ Siglec-7^+^ myeloid DCs (112).

In addition to transcriptional regulation of CD1 expression, T cell autoreactivity is controlled by the availability of self-ligands. Although determination of the repertoire of lipids bound to CD1 molecules is technically challenging, mass spectrometry analysis of lipids eluted from secreted CD1d molecules has revealed the presence of several types of phospho and sphingolipids acquired during biosynthesis (113, 114), the majority of which are non-antigenic (89). Moody and co-workers used a recently established lipidomic platform to compare self-lipids associated with all CD1 molecules and the results confirmed the ability of CD1 molecules to bind a variety of molecules (49). It is now also well established that the range of glycosphingolipids (GSL) and phospholipids expressed by cells varies amongst cell types and with cellular activation (115). TLR activation of myeloid cells has marked effect on the expression of key genes involved in GSL biosynthesis (54, 116–118), which translate in detectable biochemical changes (54, 119). This has been shown to lead to increased CD1b-restricted and iNKT cell autoreactivity (54, 116–118).

## MR1 and MAIT Cells

The MHC-related molecule MR1 (8) presents antigens to a family of innate-like T cells bearing a semi-invariant TCR and known as MAIT (10). In humans, the MAIT TCR consists of the Vα7.2 TCR-α chain mostly joined to Jα33 segments (TRAJ33) and paired to a limited number of TCR-β chains (mainly TRBV6 and TRBV20).

MR1 molecules are non-polymorphic and highly conserved among mammalian species, leading to functional cross-reactivity, which is reminiscent of the species conservation in the CD1 antigen-presenting system (120). Like iNKT cells, MAIT cells are selected in the thymus by double-positive cortical thymocytes (121), but unlike iNKT cells they leave the thymus as naïve cells and complete their maturation in the periphery (122, 123). MR1 expression on peripheral B cells and the intestinal flora are crucial for MAIT cells survival, expansion, acquisition of a memory phenotype, and effector functions (9).

Due to their anatomical mucosal localization and innate-like properties with a Th1-like effector phenotype, MAIT cells are in a unique position to act as early sentinels in response to respiratory and intestinal pathogens. Indeed, they have been shown to be activated in response to a variety of bacterial and fungal infections (124, 125) and to play a role in infectious models with *BCG*, *Francisella tularensis*, *Klebsiella penumoniae* (126–128), and MTB (129). Despite the well-characterized antimicrobial activity of MAIT cells, the antigens bound to MR1 remained for a long time elusive, until a major breakthrough in 2012 demonstrated that MR1 molecules present vitamin B2 metabolites to MAIT cells (11). These vitamins are not produced by mammals, hence they can be considered as molecular signatures of microbial infection. Consistently, microbes lacking the ability to synthesize riboflavins (such as *Streptococcus pyogenes* or *Enterococcus faecalis*) are unable to induce MR1-dependent MAIT cell activation (11).

Like iNKT cells and γδ cells, however, MAIT cells can also be activated in a TCR/MR1-independent manner, through the stimulatory activity of IL-12 and IL-18 secreted by activated APCs (130). Hence, it is possible that MAIT cells may play an immunoregulatory role also during infections with viruses and with bacteria lacking the riboflavin synthetic pathway or in sterile inflammation.

### MR1 ligands

MR1 molecules are ubiquitously expressed, although barely detectable at the cell surface (131), unless cells are incubated with vitamin ligands that increase MR1 expression (11, 132, 133). Two types of vitamin ligands have been described, stimulatory (riboflavin intermediates) and not (folic acid derivatives). Both classes of ligands have been shown to stabilize MR1 molecules, covalently binding through a Schiff base complex; however, crystallographic studies revealed that TCR recognition is exquisitely sensitive to the ribityl moiety present only in the riboflavin derivatives (134). To date, two classes of stimulatory riboflavins are known, ribityllumazine and pyrimidines [more powerful agonists, but highly unstable unless trapped by MR1 molecules (135)]. While initially the ribityllumazine rRL-6-CH_2_OH was the bacterial ligand (from *Salmonella enterica serovar Typhimurium* supernatants) thought to bind to MR1 (11), subsequent elegant studies with Gram^+^ (*Lactococcus lactis*) and Gram^−^ (*E. coli*) bacterial strains defective for key enzymes in the riboflavin synthesis pathway unveiled the intermediate compound, 5-A-RU (5-amino-6-d-ribitylaminouracil) as the key precursor for pyrimidines and ribityllumazines (133, 135). Bacterial-derived 5-A-RU itself is not stimulatory, but it reacts with bacterial or host cell-derived small glyoxal compounds to form pyrimidines, which then can condense to form ribityllumazine.

Future studies will be needed to identify the molecular mechanisms of vitamin antigen presentation through MR1. For example, the relative contribution of host-derived versus bacterial-derived glyoxal compounds that react with 5-A-RU remains to be determined, as is the cellular compartment where this condensation and the subsequent MR1 loading occur. Furthermore, the observation that some non-activating ligands [Ac-6-FP (132)] can induce rapid and prolonged upregulation of MR1 molecules suggests different effects on MR1 trafficking. Finally, the currently identified ligands are all bound in an aromatic cradle in the A′ pocket of the MR1 binding groove [although with different orientations (134)], and there remains the possibility that other classes of ligands might extend in the more exposed F′ pocket.

#### MR1 Tetramers and MAIT Cell Heterogeneity

With the discovery of MR1 ligands, MR1 tetramers have been developed to characterize the MAIT cell population, previously identified solely as TRAV1.2^+^, CD161^+^ CD8^+^ cells (136). In peripheral blood, rRL-6-CH2OH-loaded MR1 tetramers bind to a population of CD3^+^ CD4^−^ CD161^+^ cells with comparable frequency to the TRAV1.2 antibody. The advantage of tetramer over antibody stainings, however, is that tetramers are able to detect MAIT cells that have downregulated CD161 expression, such as post activation or during HIV infection (137). Single cell sorting of CD161^+^ TRAV1.2^+^ cells and CD161^+^ MR1-tetramer^+^ cells and multiplex analysis of their TCR genes revealed the use of alternative rearrangements (particularly TRAJ20 and TRAJ12) in addition to the canonical TRAV1.2-TRAJ33 (136). These alternative rearrangements have also been identified by different investigators that performed deep sequencing of mRNA from MAIT cells sorted on the basis of TRAV1.2 and CD161 co-expression (138) and on MAIT populations that specifically secreted TNF-α in response to selected pathogens (139). Diversity in the CDR3β region due to amino acid additions and a diverse use of TCR-β chains (in addition to TRBV6.4 and TRBV20) have also been observed, suggesting an unexpected heterogeneity of the peripheral MAIT T cell repertoire.

Interestingly, the canonical MAIT TCRs as well as those bearing TRAJ12 and TRAJ33 segments have a conserved Tyr95 residue in the CDR3α-chain, which is essential in forming a hydrogen bond with the ribityl tail of activating ligands (134). These three TCRs also adopt a very similar docking mode on MR1–antigen complexes (132, 140–142). However, other recently described MR1-restricted TCRs lack the Tyr95α residue (139) and future studies will be required to confirm that these TCRs do indeed confer MR1-restricted reactivity and to determine the molecular details of their antigen recognition.

Furthermore, the non-canonical TCR α-chains paired almost exclusively with TRBV6.4 (136), raising the possibility that the TCR β-chain repertoire might impact antigen recognition, as observed with iNKT cells (143, 144). Indeed, structural and biophysical data have provided experimental evidence that the CDR3β loops can fine-tune the MAIT–TCR interaction and responsiveness to MR1, in an antigen-dependent manner (132).

The functional correlate of the phenotypic heterogeneity of the MAIT repertoire is currently unclear, and an interesting hypothesis is that it may be a surrogate signature of specific pathogen infections. Along these lines, Gold and co-workers reported selective use of MAIT cell TCRs in response to three different pathogens (*Mycobacterium smegmatis, Salmonella typhimurium, and Candida albicans*) in individual subjects (139). In this data set, however, no unique TCR sequence was found to be associated with individual pathogens across individuals. In these donors, functionally responsive MAIT cells for TCR sequencing were identified by TNF-α secretion, however, in the absence of blocking experiments with MR-1 antibodies it is unknown whether the responses were entirely TCR dependent or co-stimulated by cytokines. Nevertheless, these results are of interest, as they suggest that the MAIT cell TCR repertoire potentially reflects the host’s microbial exposure history because of qualitative differences in the class of antigens presented by different pathogens, and that MAIT cells could exhibit immunological memory. However, an alternative interpretation is that different subsets of MAIT cells are differentially activated by pathogens in function of their TCR-β sequence heterogeneity (and hence of their TCR affinity), according to quantitative rather than qualitative differences in antigen availability in different microbes. Consistently, recent work by Lantz and co-workers using bacteria with mutations in the riboflavin biosynthetic pathway, suggested limited MR1 ligand heterogeneity between Gram^+^ and Gram^–^ bacteria (133).

Ultimately, longitudinal studies with well-defined microbial exposures (for example *MTB*, *Salmonella typhi* or *paratyphi*) will be needed to further explore these alternative hypotheses. In addition, it will be of interest to compare the MAIT T cell repertoire in the naïve thymus, in cord blood and in adults as MAIT cells undergo antigen-driven expansion at birth (122, 123). So far, the only study that analyzed by deep sequencing sorted TRAV1.2^+^ CD161^+^ MAIT from peripheral blood of three donors after a 5-month-interval showed that the oligoclonal TCRβ repertoire is stable in the absence of infection (138).

#### MAIT Cells in Sterile Inflammation

Although the predominant role of MAIT cells is protection against infections, there is some evidence that they may be implicated in autoimmune responses. Murine transgenic MAIT cells protect from the induction and progression of experimental autoimmune encephalomyelitis, and in MR1-deficient mice, which lack MAIT cells, EAE is exacerbated (145). MAIT cell TCR sequences were identified by single-strand polymorphism analysis in autoptic material of patients with MS (146), and one study reported a decrease in the frequency of MAIT cells in the blood of MS patients, proportional with the severity and activity of the disease (147). It has been shown that IL-18 in the serum of MS patients drives MAIT cell activation and increased expression of VLA4, an integrin that mediates migration across the blood–brain barrier (148).

Reduction of MAIT cell frequencies has been reported in the small intestine of celiac disease patients (149), while an increase was observed in the inflamed mucosa of patients with inflammatory bowel disease, with a clear activated phenotype (150). In both disease settings, changes in frequency and phenotype of tissue resident and circulating MAIT cells might be driven by compromised gut barrier function and bacterial overgrowth, as also observed during HIV infection (137, 151).

Mucosal-associated invariant T cells have been identified amongst the IL-17-producing cells in psoriatic skin, although percentages were not significantly different compared to healthy skin (152). It is currently unclear whether MAIT cells are activated in the psoriatic skin via microbial ligands or as a result of the general inflammation. Finally, it has been reported that the frequency of peripheral blood CD8 and double-negative (DN) MAIT cells is reduced in lupus and rheumatoid arthritis (RA) patients, with an accumulation of MAIT cells in the synovial fluid in RA (153). This reduction was more pronounced in patients with highly active disease. Also the capacity of MAIT to secrete IFN-γ was reduced in response to both bacterial and PMA/ionomycin stimulation, although this was shown to be unrelated to their increased expression of PD-1 (153).

While enumeration of MAIT frequencies on the basis of the sole expression of Vα7.2 and CD161 might lead to some preliminary interesting observations, further studies will need to include MR1-tetramer staining or qPCR analysis of the invariant TCR, to avoid underestimation of frequencies, as CD161 is often down-modulated following activation (154). Furthermore, the lack of a suitable animal model, due to very low frequency of MAIT cells in inbred laboratory mice may hinder the understanding of the functional relevance of the above phenotypic analysis.

Recent results have brought MAIT cells to the center stage in chronic inflammatory settings associated with obesity and diabetes, possibly as a consequence of the altered composition of the gut microbiota in both diseases (155). The frequency of circulating MAIT cells, as determined by both MR1 tetramer staining and TRAV7.2 and CD161 staining, was significantly reduced as compared to healthy controls. The remaining MAIT cells showed a phenotype consistent with activation (upregulation of CD25 and CD69) and an inflammatory cytokine bias (higher secretion of IFN-γ, IL-2, IL-7, granzyme B). Conversely, MAIT cells were increased in subcutaneous and omental adipose tissue as compared to the blood, suggesting preferential tissue recruitment. Adipose tissue MAIT cells also secreted more IL-17 in obese as compared to lean patients. Interestingly, the authors observed an attenuation of MAIT cell abnormalities after weight loss following bariatric surgery. It is possible that IL-7 produced by adipose tissue stromal cells facilitates MAIT cell activation, as previously observed in the liver (156). In addition to cytokines, changes in gut microbiota and permeability might release a variety of bacterial ligands, which could react with increased endogenous levels of methylglyoxals to form MAIT cell agonists.

### MAIT interactions with APCs

As mentioned before, MR1 is ubiquitously transcribed, although cell surface expression is very low and it is only transiently upregulated following infection or incubation with some of the synthetic ligands (27, 131–133). Upon infection, MAIT cells can be activated in a MR1-dependent way by a variety of cells, including DC, macrophages, epithelial cells, and fibroblasts (124, 125). By secreting a plethora of regulatory cytokines (138), activated MAIT like iNKT cells may be able to modulate the antimicrobial function of other cells. Likewise, by secreting chemokines like CCL4 they can recruit NK, monocytes, and other inflammatory cells to infected tissues (138). Furthermore, it is likely that MAIT cell activation will provide an early source of IFN-γ during infections, facilitating the development of Th1 immunity, as described for NK cells (157), γδ (87), and iNKT cells (158). However, it is currently unknown whether MAIT cells are capable of inducing effective DC maturation *in vivo*, and if so the relative contribution of cytokines and CD40–CD40L interactions, which are key for DC licensing by CD1-restricted cells (103).

Post-natal MAIT cell expansion depends on bacterial flora and B cells (9). *In vitro*, primary B cells and EBV-transformed B cell lines have been shown to induce MAIT cell activation in an MR1-dependent manner following infection with commensal or pathogenic intestinal bacteria (159). Lack of titration of MAIT cell activation and reduced stimulation by paraformaldehyde fixed B cells, however, suggest a possible contribution of soluble factors, which was not addressed. Consistent with this, IFN-γ secretion by activated MAIT cells was only partially blocked by the anti MR1 antibody 26.5.

Finally, despite known expression of the CD161 ligand LLT1 by activated B cells and DC (160, 161), its role in modulating MAIT cell reactivity remains to be addressed, and could be of relevance considering the profound CD161 downmodulation observed with MAIT cell activation (154).

## Concluding Remarks

Our current understanding of innate-like T cell populations has been widened in the past few years by few key technological advances, such as the identification of novel agonists and the capacity to refold antigen-presenting molecules to generate tetramers to enumerate qualitatively and quantitative these cells in health and disease settings. Biophysical and crystallographic studies, coupled with extensive mutagenesis, have elucidated the fine molecular details of antigen recognition, highlighting the existence of conserved (for iNKT, MAIT, and GEM T cells) and more variable (for CD1a and CD1c-restricted T cells) TCR footprints over the cognate antigen-presenting molecules (40). In the future, a better understanding of the fine details of antigen presentation through MR-1 may open new avenues aimed at therapeutically harnessing MAIT cells in promoting the cross talk between the innate and adaptive arms of the immune system. Given the higher frequencies of MAIT cells over iNKT cells in humans, and their enrichment at mucosal sites, MAIT cell agonists might prove effective adjuvants to promote mucosal immune responses.

Finally, functional and phenotypical enumeration of MAIT cells and GEM T cells with tetramers may become a valuable immuno-monitoring tool. For example, it has been shown that MAIT cell frequencies are reduced in the blood of individuals with active MTB infection but they normalize after therapy (154), hence it should be explored whether they could be considered a marker of disease status, possibly to identify individuals at risk of progression to clinically active disease. Additional population of invariant T cells are being discovered by next-generation sequencing of the TCR-α chain repertoire (162) and may be used to probe antigenic exposure at a population level.

## Conflict of Interest Statement

The authors declare that the research was conducted in the absence of any commercial or financial relationships that could be construed as a potential conflict of interest.
